# A Randomized, Phase 3 Trial of Naltrexone SR/Bupropion SR on Weight and Obesity-related Risk Factors (COR-II)

**DOI:** 10.1002/oby.20309

**Published:** 2013-02-14

**Authors:** Caroline M Apovian, Louis Aronne, Domenica Rubino, Christopher Still, Holly Wyatt, Colleen Burns, Dennis Kim, Eduardo Dunayevich, for the COR-II Study Group

**Affiliations:** 1 Section of Endocrinology, Diabetes and Nutrition, Department of Medicine, Boston University School of MedicineBoston, Massachusetts, USA; 2 Weill Cornell Medical CollegeNew York, New York, USA; 3 Washington Center for Weight ManagementArlington, Virginia, USA; 4 Geisinger Health Care SystemDanville, Pennsylvania, USA; 5 University of Colorado DenverDenver, Colorado, USA; 6 Orexigen Therapeutics, Inc.La Jolla, California, USA

## Abstract

**Objective:**

To examine the effects of naltrexone/bupropion (NB) combination therapy on weight and weight-related risk factors in overweight and obese participants.

**Design and Methods:**

CONTRAVE Obesity Research-II (COR-II) was a double-blind, placebo-controlled study of 1,496 obese (BMI 30-45 kg/m^2^) or overweight (27-45 kg/m^2^ with dyslipidemia and/or hypertension) participants randomized 2:1 to combined naltrexone sustained-release (SR) (32 mg/day) plus bupropion SR (360 mg/day) (NB32) or placebo for up to 56 weeks. The co-primary endpoints were percent weight change and proportion achieving ≥5% weight loss at week 28.

**Results:**

Significantly (*P* < 0.001) greater weight loss was observed with NB32 versus placebo at week 28 (−6.5% vs. −1.9%) and week 56 (−6.4% vs. −1.2%). More NB32-treated participants (*P* < 0.001) experienced ≥5% weight loss versus placebo at week 28 (55.6% vs. 17.5%) and week 56 (50.5% vs. 17.1%). NB32 produced greater improvements in various cardiometabolic risk markers, participant-reported weight-related quality of life, and control of eating. The most common adverse event with NB was nausea, which was generally mild to moderate and transient. NB was not associated with increased events of depression or suicidality versus placebo.

**Conclusion:**

NB represents a novel pharmacological approach to the treatment of obesity, and may become a valuable new therapeutic option.

## Introduction

As the prevalence of obesity increases among adults and children (1), obesity-related health complications are predicted to drive the first decrease in life expectancy in modern history (2). Weight loss of 5-10% is associated with reduced metabolic and cardiovascular risk (3); however, many individuals are not able to achieve or maintain this weight loss with diet and exercise alone (3-4).

Targeting multiple pathways often enhances pharmacotherapeutic efficacy, such as in the treatment of hypertension and type 2 diabetes (5-6). Many CNS pathways influence weight (7), making combination agents a promising pharmacotherapeutic approach for weight loss. The naltrexone/bupropion (NB) combination was developed based on preclinical evidence that NB has complementary actions in the CNS that reduce food intake (7-9). Bupropion stimulates hypothalamic pro-opiomelanocortin (POMC) neurons, with downstream effects to reduce food intake and increase energy expenditure. Naltrexone blocks opioid receptor-mediated POMC auto-inhibition, augmenting POMC firing in a synergistic manner (9). Given the known effects of naltrexone and bupropion on addiction (alcohol (10) and nicotine (11), respectively), NB is hypothesized to induce weight loss through sustained modulation of CNS reward pathways.

Initial Phase 2 studies in obese adults indicated that combined naltrexone and bupropion resulted in greater weight loss than the additive effects of the individual components (12). Here, we present the results of the Contrave® (proposed commercial name for NB) Obesity Research-II (COR-II) trial, a Phase 3 study conducted to evaluate the efficacy and safety of an SR formulation of NB for up to 56 weeks in overweight and obese participants. COR-II is one of four Phase 3 trials evaluating the safety and efficacy of NB for the treatment of obesity (13-14).

## Methods and Procedures

### Study design and participants

This was a Phase 3 randomized, parallel-arm, double-blind, placebo-controlled, 56-week study. Participants were 18-65 years with a BMI 30-45 kg/m^2^, or a BMI 27-45 kg/m^2^ and controlled hypertension and/or dyslipidemia. The study was conducted at 36 US private or institutional practices between December 2007 and June 2009.

Exclusion criteria included diabetes; significant vascular, hepatic, or renal disease; weight change of >4 kg within 3 months prior to randomization; history of seizures or serious psychiatric illness. Additional eligibility details are available in the Supporting Information Section 1.

All participants provided written informed consent, and the protocol was approved by an institutional review board for each institution. The study complied with Good Clinical Practice standards and the Declaration of Helsinki. (15) A data safety monitoring committee performed regular reviews of interim safety analyses.

### Procedures and endpoints

**FIGURE 1:**
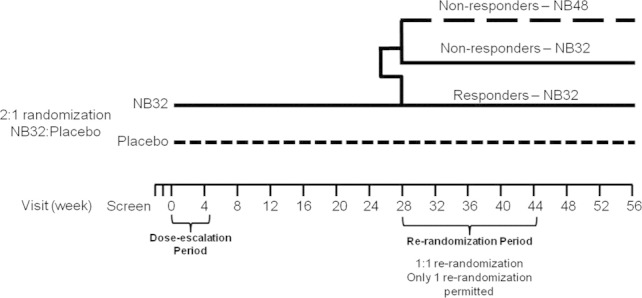
Following screening, participants were randomized via a centrally administered interactive voice response system in a 2:1 ratio, stratified by study site, to receive a combined oral formulation of 32 mg/day naltrexone SR + 360 mg/day bupropion SR (NB32) or matching placebo, administered in divided doses twice daily. Naltrexone was initiated at one-eighth or one-quarter of the maintenance dose and bupropion was initiated at one-quarter of the maintenance dose; doses were escalated linearly over the first 3-4 weeks, and the maintenance dose was reached by the start of week 5. To evaluate the efficacy and safety of a dose increase in participants with suboptimal response, NB32 participants with <5% weight loss at visits between weeks 28 and 44 inclusive were re-randomized (double-blind, 1:1 ratio) to continue receiving NB32 or escalate to NB48 (48 mg/day naltrexone SR + 360 mg/day bupropion SR) for the remainder of the study. Study visits occurred at baseline (week 0) and every 4 weeks thereafter.

Following screening, participants were randomized via a centrally administered interactive voice response system in a 2:1 ratio, stratified by study site, to receive a combined oral formulation of 32 mg/day naltrexone SR + 360 mg/day bupropion SR (NB32) or matching placebo, administered in divided doses, twice daily (Figure 1). Study drug was escalated weekly over the first 3-4 weeks; full dose was reached by the start of week 5. To evaluate the efficacy and safety of a dose increase in participants with suboptimal response, NB32 participants with <5% weight loss at visits between weeks 28 and 44 inclusive were re-randomized (double-blind, 1:1 ratio) to continue receiving NB32 or escalate to NB48 (48 mg/day naltrexone SR + 360 mg/day bupropion SR) for the remainder of the study. Study visits occurred at baseline and every 4 weeks. At baseline, 12, 24, 36, and 48 weeks, participants received instructions to follow a hypocaloric diet (500 kcal/day deficit) and increase physical activity, and behavioral modification advice. Weight and vital signs were measured at each visit.

Efficacy questionnaires included the Impact of Weight on Quality of Life (IWQOL)-Lite (16) and the Control of Eating Questionnaire (COEQ). (17) The COEQ uses 100-mm visual analog scales to assess appetite, food craving, eating behavior, and mood over the 7 days prior to questionnaire administration.

The two co-primary efficacy endpoints were the percent change in weight and the proportion of participants with ≥5% weight loss at week 28, with secondary endpoint analyses of these measures at week 56. Additional secondary endpoints included the proportion of participants with ≥10% weight loss and changes in markers of cardiometabolic risk, participant-reported measures of food cravings and control of eating, and weight-related quality of life at week 28. Prespecified tertiary endpoints included the above measures at week 56. Exploratory analyses included percent change in weight from re-randomization and baseline to week 56 for participants re-randomized to NB32 or NB48.

Safety assessments included evaluation of treatment-emergent adverse events, vital signs, electrocardiograms, and clinical laboratory measures. Depressive symptoms were evaluated at each visit using the Inventory of Depressive Symptomatology–Self Rated (IDS-SR) (18). In addition, 182 participants at nine study sites were enrolled in a substudy in which ambulatory blood pressure and heart rate were measured hourly over 24-hour periods at baseline, week 24, and week 52; 180 met criteria for inclusion into the substudy analysis set (121 NB and 59 placebo).

### Statistical analyses

To obtain the targeted number of participant-exposures at one year, it was estimated that 1,000 participants would need to be randomized to NB32, with an assumed 40% attrition rate (12), with a 99%, 81%, and 70% chance that ≥1 adverse event would be observed at a true frequency of 1/100, 1/250, and 1/500, respectively. It was estimated that 1,500 randomized participants (2:1 ratio) would provide 99% power to detect a statistically significant difference in mean percent weight loss of ≥5%, and a 14% difference in the proportion of participants with ≥5% weight loss between NB32 and placebo. Power estimates were determined using a two-sample *t*-test for mean percent weight loss and a two-sample continuity-corrected chi-square test for the proportion of participants with ≥5% weight loss using a two-sided significance level of 5%.

Unless otherwise specified, efficacy analyses were performed on a prespecified modified intent-to-treat (mITT) analysis population composed of all randomized participants with a baseline weight and ≥1 post-baseline weight on study drug (+1 day post-last dose). Missing data were imputed by carrying forward the last observation on study drug (LOCF). In prespecified week 56 efficacy analyses of NB32, data for participants re-randomized to NB32 were double-weighted and participants re-randomized to NB48 were excluded. The safety population included all randomized participants who took ≥1 tablet of study drug and had ≥1 investigator contact/assessment at any time after the start of study treatment. The ambulatory blood pressure monitoring substudy population included all participants who were randomized in the substudy, had a baseline measurement, received treatment, and had at least one investigator contact/assessment after the start of study treatment.

Four sensitivity analyses were conducted for the body weight endpoints: 1) BOCF: all randomized participants, where the baseline observation was carried forward for participants who discontinued study drug prior to weeks 28 or 56; 2) ITT-MMRM: participants with a post-baseline weight measurement (on or off study drug) and used a repeated measures linear mixed-effects model, with random participant effects and fixed class effects for treatment, time, study center, and treatment-by-time interaction, with baseline as a covariate; 3) mITT-LOCF unweighted: participants in the mITT population and grouped all NB32/48-treated participants together regardless of re-randomization status; and 4) completers: participants who completed 28 or 56 weeks of treatment.

General linear models (analysis of covariance [ANCOVA]) including terms for treatment and study center, with baseline values as a covariate, were used to analyze the co-primary and continuous secondary endpoints. Categorical endpoints were analyzed using a logistic regression model including treatment and study center as main effects and baseline value as covariate. To maintain the family-wise type I error rate at 5%, secondary endpoints were analyzed in a predetermined sequence only after both co-primary endpoints achieved statistical significance, beginning with the percentage of weight loss at week 56 and the proportion of participants with ≥5% weight loss at week 56, and continuing with the proportion of participants with ≥10% weight loss at week 28, and the change from baseline to week 28 in the following: waist circumference, high-density lipoprotein (HDL)-cholesterol, triglycerides, IWQOL-Lite total score, hsCRP, insulin, glucose, HOMA-IR (insulin resistance, derived from the homeostasis model assessment), COEQ control of eating question (#19), low-density lipoprotein (LDL)-cholesterol, systolic blood pressure, diastolic blood pressure, and IDS-SR total score. Formal testing was conducted in a step-down manner until any endpoint failed to reach *P* < 0.05, after which the nominal *P*-values are reported and findings are considered exploratory. To control for skewness, analyses for triglycerides, hsCRP, insulin, and HOMA-IR were log10 transformed prior to running the ANCOVA models. The percent change from baseline was calculated by back-transforming the LS geometric mean minus one. All statistical analyses were performed using Windows SAS version 9.1. Continuous endpoints are provided as LS mean ± SE unless otherwise indicated.

## Results

**FIGURE 2:**
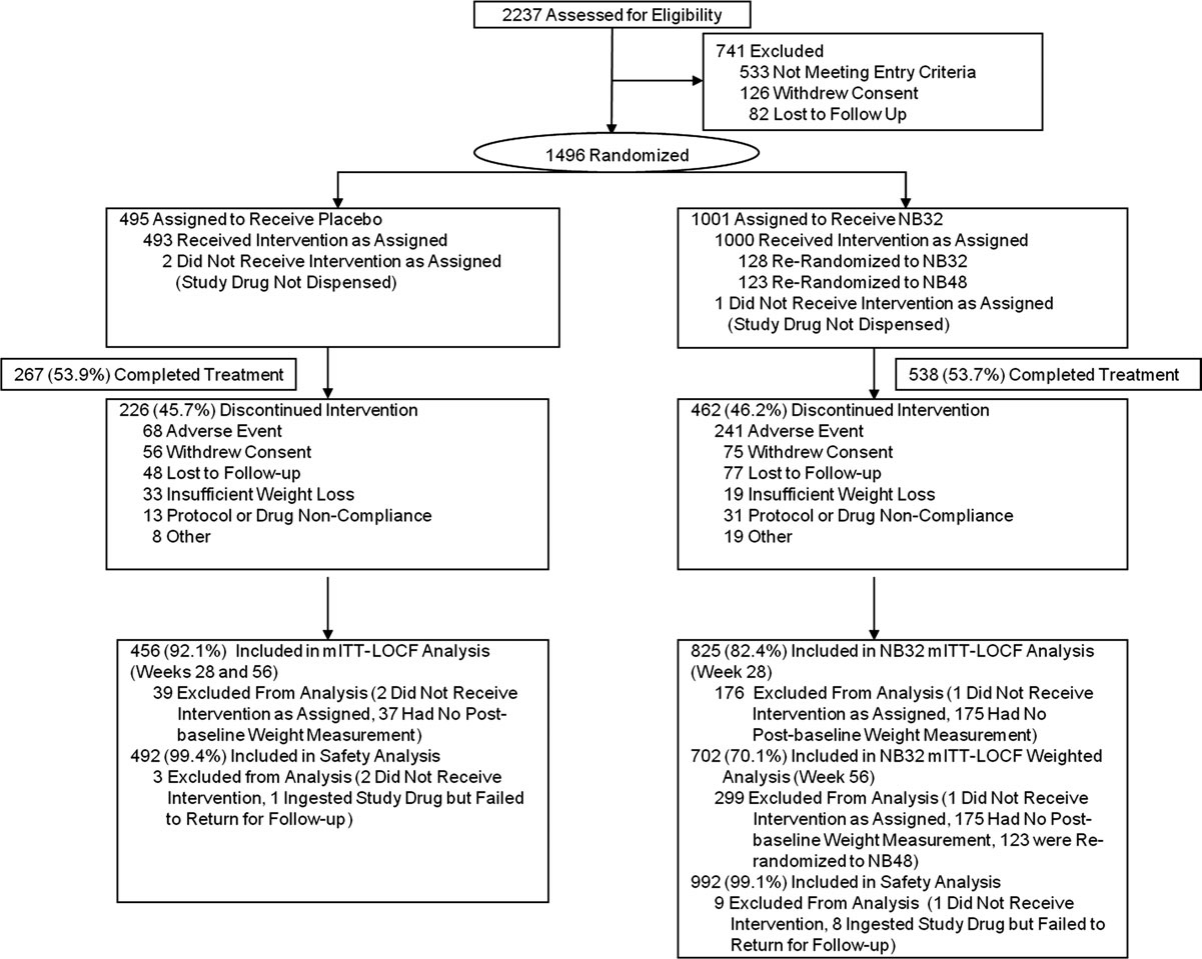
Participant flow chart.

Of the 1,496 participants randomized to double-blind treatment, 54% of participants in each treatment group completed 56 weeks of treatment (Figure 2). More NB-treated participants discontinued because of an adverse event (*P* < 0.001), whereas more placebo-treated participants discontinued because of insufficient weight loss (*P* < 0.001) and withdrawal of consent (*P* < 0.05). Discontinuations in both groups occurred most frequently during the first 8 weeks of the study, with more discontinuations, particularly because of AEs, occurring with NB (Table 4). Visit-wise discontinuation rates during the remainder of the study were generally similar between groups. Demographics and baseline characteristics were similar between treatment groups (Table 1).

**TABLE 1:** Demographics and baseline characteristics

Demographic/characteristic^a^	Placebo	NB^b^
	*N* = 495	*N* = 1001
Age, y	44.4 ± 11.4	44.3 ± 11.2
Gender (% female)	84.8	84.6
Race (% White/Black/Other)	84/15/2^c^	83/13/3^c^
Weight, kg	99.2 ± 15.9	100.3 ± 16.6
BMI, kg/m^2^	36.1 ± 4.3	36.2 ± 4.5
Hypertension, %^d^	21.4	21.2
Dyslipidemia, %^e^	53.1	55.9

a Data are mean ± SD or % of participants for the Randomized population.

b NB group includes all participants randomized to NB32 at baseline, regardless of re-randomization status.

c Percentages may not add up to 100 because of rounding.

d Diagnosed at baseline with hypertension or prescribed antihypertensive concomitant medications.

e Diagnosed at baseline with dyslipidemia, hypercholesterolemia, hypertriglyceridemia, hyperlipidemia, low HDL-cholesterol or with at least one of the following values prior to first dose of study drug: triglycerides ≥200 mg/dL, LDL-cholesterol ≥160 mg/dL, total cholesterol ≥240 mg/dL, HDL-cholesterol <40 mg/dL.

**FIGURE 3:**
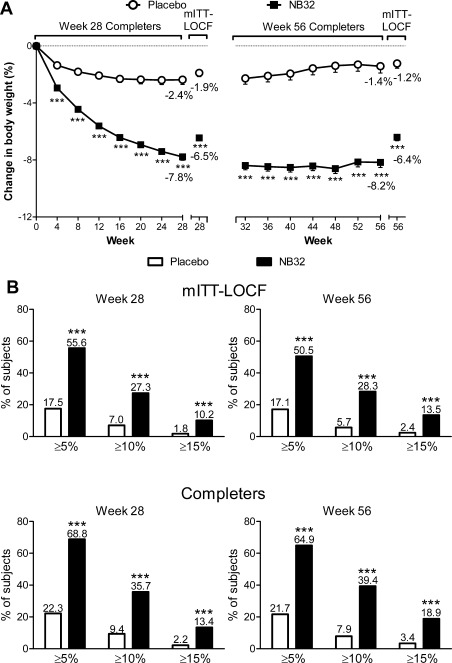
A) Percent weight loss (observed; LS mean ± SE) by visit in the week 28 and 56 completers populations (NB32 data are weighted for weeks 32-56), and percent weight loss for the week 28 and 56 mITT-LOCF populations. ***P < 0.001 for NB32 vs. Placebo. B) Categorical weight loss in week 28 and 56 mITT-LOCF and Completers populations. ***P < 0.001 for NB32 vs. Placebo. In both panels, week 56 data for NB32 are weighted as described in the *Statistical analyses* section.

In the mITT-LOCF population, weight loss was significantly greater for NB32 versus placebo at week 28 (−6.5% vs. −1.9%; *P* < 0.001). Weight loss was maintained with continued double-blind treatment in the NB32 group through week 56 (−6.4% vs. −1.2; *P* < 0.001; Figure 3A). NB32 was associated with a significantly larger proportion of participants achieving ≥5%, ≥10%, and ≥15% weight loss in the mITT-LOCF population versus placebo at weeks 28 and 56 (Figure 3B). Participants completing 56 weeks of treatment had more pronounced weight loss (−8.2% NB32 vs. −1.4% placebo; *P* < 0.001) and were more likely to achieve ≥5%, ≥10%, and ≥15% weight loss than the mITT-LOCF population (Figure 3). Using different imputation methods and populations, greater weight loss was consistently demonstrated with NB compared to placebo (Table 2). The percent weight change from the time of re-randomization to week 56 (mITT-LOCF) for suboptimal responders re-randomized to NB32 (*n* = 124) versus NB48 (*n* = 120) was similar (+1.0% vs. +0.6%; *P* = 0.35), as was the percent weight change from study baseline to week 56 (mITT-LOCF; −1.3% vs. −1.1%; *P* = 0.77).

**TABLE 2:** Change in body weight at weeks 28 and 56 by study population

	Week 28			Week 56		
		
Measure^a^	Placebo	NB32	*P*-value	Placebo	NB32^b^	*P*-value
**Number of participants**						
mITT-LOCF	456	825		456	702^c^	
ITT-MMRM	473	928		473	805^c^	
Completers	319	619		267	434^c^	
BOCF	495	1001		495	878^c^	
mITT-LOCF, unweighted^d^				456	825	
**Body weight, %**						
mITT-LOCF	−1.9 ± 0.3	−6.5 ± 0.2	<0.001^e^	−1.2 ± 0.3	−6.4 ± 0.3	<0.001^f^
ITT-MMRM	−2.0 ± 0.3	−6.6 ± 0.2	<0.001	−0.9 ± 0.4	−6.3 ± 0.3	<0.001
Completers	−2.4 ± 0.3	−7.8 ± 0.2	<0.001	−1.4 ± 0.5	−8.2 ± 0.4	<0.001
BOCF	−1.5 ± 0.3	−4.8 ± 0.2	<0.001	−0.8 ± 0.3	−4.4 ± 0.2	<0.001
mITT-LOCF, unweighted^d^				−1.2 ± 0.3	−6.3 ± 0.2	<0.001
**Body weight, kg**						
mITT-LOCF	−2.0 ± 0.3	−6.3 ± 0.2	<0.001	−1.3 ± 0.3	−6.2 ± 0.2	<0.001
ITT-MMRM	−2.1 ± 0.3	−6.5 ± 0.2	<0.001	−1.0 ± 0.4	−6.2 ± 0.3	<0.001
Completers	−2.5 ± 0.3	−7.6 ± 0.2	<0.001	−1.5 ± 0.5	−7.9 ± 0.3	<0.001
BOCF	−1.6 ± 0.3	−4.7 ± 0.2	<0.001	−0.8 ± 0.3	−4.3 ± 0.2	<0.001
mITT-LOCF, unweighted^d^				−1.3 ± 0.3	−6.3 ± 0.2	<0.001
**Participants with ≥5% weight loss**						
mITT-LOCF	17.5%	55.6%	<0.001^e^	17.1%	50.5%	<0.001^f^
Completers	22.3%	68.8%	<0.001	21.7%	64.9%	<0.001
BOCF	13.9%	42.1%	<0.001	11.7%	35.1%	<0.001
mITT-LOCF, unweighted^d^				17.1%	51.0%	<0.001
**Participants with ≥10% weight loss**						
mITT-LOCF	7.0%	27.3%	<0.001^f^	5.7%	28.3%	<0.001
Completers	9.4%	35.7%	<0.001	7.9%	39.4%	<0.001
BOCF	5.9%	21.9%	<0.001	4.2%	21.3%	<0.001
mITT-LOCF, unweighted^d^				5.7%	28.2%	<0.001
**Participants with ≥15% weight loss**^g^						
mITT-LOCF	1.8%	10.2%	<0.001	2.4%	13.5%	<0.001
Completers	2.2%	13.4%	<0.001	3.4%	18.9%	<0.001
BOCF	1.4%	8.1%	<0.001	2.0%	10.2%	<0.001
mITT-LOCF, unweighted^d^				2.4%	13.5%	<0.001

BOCF, baseline observation carried forward; ITT, intent-to-treat; LOCF, last observation carried forward; mITT, modified intent-to-treat; MMRM, repeated measures linear mixed-effects model.

a Data are LS mean ± SE or percentage of participants (%).

b Unless otherwise indicated, week 56 data for NB32 are weighted as described in the *Statistical analyses* section.

c For mITT-LOCF Week 56 analysis, 124 participants re-randomized to NB32 were double-weighted. For ITT-MMRM and BOCF week 56 analyses, 128 participants re-randomized to NB32 were double-weighted. For Completers week 56 analysis, 107 participants re-randomized to NB32 were double-weighted.

d The unweighted sensitivity analysis pooled all NB participants together for change from baseline to week 56 endpoint analyses regardless of re-randomization status.

e Co-primary endpoints.

f Endpoints that were significant according to the prespecified sequential closed testing procedure conducted to control for multiple comparisons.

g Exploratory analysis.

NB32 resulted in improvements in various cardiometabolic parameters, including waist circumference, triglycerides, and HDL versus placebo at week 28 (Table 3). NB32 was also associated with reduced LDL, as well as reduced fasting insulin and HOMA-IR. In most cases, improvements in secondary endpoints were maintained at week 56. At week 28, NB32 was associated with improvement in total IWQOL-Lite score versus placebo (*P* < 0.001) and greater improvements for NB32 versus placebo were observed in the physical function, self-esteem, and sexual life subscales (*P* < 0.01; Supporting Information Section 2). Greater improvements in IWQOL-Lite total score and subscale scores were maintained through week 56.

**TABLE 3:** Changes in secondary and additional endpoints

	Week 28			Week 56		
		
Measure^a^	Placebo *N* = 456	NB32 *N* = 825	*P*-value	Placebo *N* = 456	NB32^b^*N* = 702	*P*-value
Waist circumference, cm						
Baseline	108.9 ± 11.7	109.3 ± 11.9		108.6 ± 11.8	109.0 ± 11.8	
Change	−2.7 ± 0.4	−6.2 ± 0.3	<0.001^c^	−2.1 ± 0.5	−6.7 ± 0.3	<0.001
Triglycerides, mg/dL^d^						
Baseline	113.4 ± 1.6	119.0 ± 1.6		112.8 ± 1.6	118.9 ± 1.6	
Percent change (95% CI)	−1.4% (−5.0%, +2.4%)	−7.3% (−9.8%, −4.8%)	0.007^c^	−0.5% (−4.5%, +3.7%)	−9.8% (−12.4%, −7.1%)	<0.001
HDL-cholesterol, mg/dL						
Baseline	51.4 ± 13.1	51.4 ± 13.3		51.6 ± 12.9	51.8 ± 13.6	
Change	−1.4 ± 0.4	+1.2 ± 0.3	<0.001^c^	−0.9 ± 0.5	+3.6 ± 0.4	<0.001
LDL-cholesterol, mg/dL						
Baseline	117.1 ± 32.6	119.8 ± 30.2		116.8 ± 32.9	120.5 ± 30.2	
Change	0.0 ± 1.3	−4.4 ± 0.9	0.004	−2.1 ± 1.3	−6.2 ± 0.9	0.008
hsCRP, mg/L^d^						
Baseline	3.7 ± 2.7	3.9 ± 2.8		3.7 ± 2.8	3.8 ± 2.8	
Percent change (95% CI)	−1.1% (−9.1%, +7.5%)	−9.4% (−14.8%, −3.6%)	0.091	−8.3% (−17.2%, +1.6%)	−28.8% (−33.9%, −23.3%)	<0.001
Fasting blood glucose, mg/dL						
Baseline	94.2 ± 10.4	94.8 ± 11.2		94.2 ± 10.4	95.0 ± 11.3	
Change	−1.7 ± 0.5	−2.1 ± 0.4	0.544	−1.3 ± 0.6	−2.8 ± 0.5	0.051
Fasting insulin, μIU/mL^d^						
Baseline	10.7 ± 1.9	11.4 ± 1.9		10.7 ± 1.9	11.4 ± 1.9	
Percent change (95% CI)	−0.5% (−6.5%, +5.9%)	−14.1% (−17.9%, −10.2%)	<0.001	+3.5% (−3.8%, +11.2%)	−11.4% (−15.9%, −6.6%)	<0.001
HOMA-IR^d^						
Baseline	2.5 ± 2.0	2.7 ± 2.0		2.5 ± 2.0	2.7±2.0	
Percent change (95% CI)	−4.2% (−10.4%, +2.6%)	−16.4% (−20.4%, −12.3%)	<0.001	+1.2% (−6.5%, +9.6%)	−13.8% (−18.6%, −8.7%)	<0.001
IWQOL-Lite total score^e^						
Baseline	72.9 ± 15.7	72.0 ± 17.4		73.0 ± 15.9	71.9 ± 17.1	
Change	+6.2 ± 0.6	+9.9 ± 0.4	<0.001^c^	+6.4 ± 0.6	+10.9 ± 0.5	<0.001
COEQ, control of eating^f^						
Baseline	62.0 ± 23.5	61.9 ± 24.1		62.0 ± 23.5	62.8 ± 23.9	
Change	−11.1 ± 1.1	−18.3 ± 0.9	<0.001	−11.3 ± 1.2	−15.9 ± 0.9	0.002
Systolic blood pressure, mm Hg						
Baseline	118.2 ± 10.5	118.1 ± 10.0		118.2 ± 10.5	117.9 ± 10.0	
Change	−1.2 ± 0.4	−0.9 ± 0.3	0.556	−0.5 ± 0.4	+0.6 ± 0.3	0.039
Diastolic blood pressure, mm Hg						
Baseline	76.8 ± 7.0	76.8 ± 7.0		76.8 ± 7.0	76.7 ± 7.0	
Change	−0.7 ± 0.3	+0.2 ± 0.2	0.017	+0.3 ± 0.3	+0.4 ± 0.2	0.847
IDS-SR total score^g^						
Baseline	6.9 ± 5.3	7.2 ± 6.0		6.9 ± 5.3	7.0 ± 5.9	
Change	−0.3 ± 0.2	−0.2 ± 0.2	0.844	−0.5 ± 0.3	−0.3 ± 0.2	0.689

COEQ, Control of Eating Questionnaire; CI, confidence interval; HDL, high-density lipoprotein; HOMA-IR, homeostasis model assessment of insulin resistance; hs-CRP, high-sensitivity C reactive protein; IDS-SR, Inventory of Depressive Symptomatology -Self Rated; IWQOL-Lite, Impact of Weight on Quality of Life–Lite version; LDL, low-density lipoprotein.

SI Conversion Factors: To convert values for triglycerides to mmol/L, multiply by 0.0113. To convert values for HDL and LDL cholesterol to mmol/L, multiply by 0.0259. To convert values for glucose to mmol/L, multiply by 0.0555. To convert values for insulin to pmol/L, multiply by 6.945.

a Data are for the mITT-LOCF population, where the last observation on study drug was carried forward. Unless otherwise specified, baseline values are mean ± SD and change values are LS mean ± SE.

b Week 56 data for NB32 are weighted as described in the *Statistical analyses* section.

c Secondary endpoints that were significant according to the prespecified sequential closed testing procedure conducted to control for multiple comparisons.

d Baseline values are geometric mean ± SD; percent change values are LS mean (95% CI); *P*-values are based on log transformed values.

e IWQOL-Lite total score is based on a scale from 0 to 100 where a score of 72-79 indicates moderate impairment.

f COEQ question #19: Generally, how difficult has it been to control your eating? (scoring: 0 = not at all difficult; 100 = extremely difficult)

g IDS-SR total score is based on 30 items. The total score can range from 0-84, with 0 being no depressive symptoms and 84 being severe depressive symptoms. A total score ≤13 indicates no depression.

Exploratory analyses of the COEQ revealed an association between NB32 and improved control of eating and reduced food craving, as well as other items. Generally, the greatest reductions in COEQ items occurred early in trial. However, greater improvements (*P* < 0.05) were observed at all time points for NB32 versus placebo in COEQ items 9, 11, and 19 indicating reduced frequency of food cravings as well as reduced difficulty in resisting food cravings and controlling eating (Supporting Information Section 3).

Mean systolic and diastolic blood pressure tended to remain within approximately 1 mm Hg of baseline values in both placebo- and NB-treated subjects throughout the study; mean blood pressure was slightly lower with placebo (Table 3 [mITT-LOCF population] and Table 4 [safety population]). Pulse rate was unchanged with placebo and a small, approximately 1 bpm increase was observed with NB. NB32 and placebo arms demonstrated similar categorical changes in blood pressure and pulse rate (Supporting Information Section 4), although NB32 was generally associated with a numerically greater proportion of outliers. The relationship of greater blood pressure reduction with greater weight loss was evident for both treatment groups (Supporting Information Section 5).

**TABLE 4:** Adverse events and safety endpoints

	Placebo	NB
	*N* = 492	*N* = 992
Participants (%) reporting any adverse event	75.2	85.9
Nausea	6.9	29.2*
Constipation	7.1	19.1*
Headache	8.7	17.5*
Insomnia	6.7	9.8
Dry mouth	2.6	9.1*
Upper respiratory tract infection	11.2	8.7
Vomiting	2.0	8.5*
Nasopharyngitis	8.1	8.3
Dizziness	3.7	6.9*
Diarrhea	3.7	5.5
Sinusitis	7.1	5.1
Arthralgia	5.7	3.8
Bronchitis	5.1	1.4*
Participants (%) reporting any psychiatric adverse event	15.2	20.7*
Insomnia	6.7	9.8
Anxiety	4.3	4.8
Depression	1.6	1.3
Sleep disorder	0.8	1.1
Participants (%) reporting any adverse event leading to discontinuation	13.8	24.3*
Nausea	0.2	6.0*
Headache	0.8	2.6*
Depression	1.2	0.5
Safety Endpoints		
Systolic blood pressure, mm Hg		
Baseline	118.3 ± 10.5	118.2 ± 10.1
Change from baseline to week 56	−0.4 ± 0.4	+0.2 ± 0.3
Diastolic blood pressure, mm Hg		
Baseline	76.8 ± 7.0	76.8 ± 7.0
Change from baseline to week 56	+0.1 ± 0.3	0.0 ± 0.2
Pulse rate, bpm		
Baseline	71.4 ± 8.5	71.2 ± 8.6
Change from baseline to week 56	−0.3 ± 0.3	+0.8 ± 0.2*

Safety analysis set. NB group includes all participants in the safety analysis set randomized to NB32 at baseline, regardless of re-randomization status. Adverse events with incidence >5% in any treatment group are reported; Psychiatric adverse events with incidence >1% in any treatment group are reported; Adverse events leading to discontinuation with incidence >1% in any treatment group are reported; For vital signs, baseline values are mean ± SD, change values are LS mean ± SE (LOCF);

* *P* < 0.05 for NB vs. Placebo comparison.

In a substudy, 24-hour systolic and diastolic blood pressure and heart rate patterns were similar between NB and placebo-treated participants at baseline, week 24, and week 52. The normal circadian variation of blood pressure, including a nocturnal decrease, was maintained in both treatment groups (data not shown).

NB was associated with a greater incidence of adverse events than placebo and more participants in the NB group discontinued treatment because of an adverse event (Table 4), particularly early in the trial. The most frequent treatment-emergent adverse events were nausea, headache, and constipation. These events were mostly mild to moderate and did not result in discontinuation in most participants who experienced them. Most nausea events occurred during the dose escalation period and were transient. IDS-SR total scores were in the nondepressed range at baseline and remained so throughout the study. There were no differences between NB and placebo at endpoint on IDS-SR total score or key items measuring sadness, irritability, anxiety/tension, and suicidality. There was one event of passive suicidal ideation in an NB32-treated participant; symptoms resolved following study drug discontinuation. NB was not associated with increased incidence of treatment-emergent symptoms of depression or other mood-related adverse events.

The proportion of participants who experienced a serious adverse event was similar for NB (2.1%) and placebo (1.4%). There was one myocardial infarction in an NB-treated participant with active coronary artery disease, angina pectoris, hyperlipidemia, and hypertension. One seizure was reported for an NB-treated participant with no history of seizures. There were no clinically significant effects of NB on laboratory measures or ECG.

## Discussion

This study demonstrates that NB32 is associated with significantly greater weight loss and greater improvement in some measures of cardiometabolic risk than placebo. Greater weight loss was observed with NB32 versus placebo at the first time point (week 4) and was sustained over the 56-week trial. A greater proportion of NB32-treated participants achieved ≥5%, ≥10%, and ≥15% weight loss versus placebo. Similar results were observed with several sensitivity analyses, demonstrating the robustness of these results. Individuals who did not attain or maintain ≥5% weight loss with NB32 did not appear to benefit from an increased naltrexone dose. NB32 treatment was also associated with improvements in many cardiometabolic parameters, including waist circumference, lipids, insulin, and insulin sensitivity.

Adverse events with NB appear consistent with the known profiles of naltrexone and bupropion, which individually have over 25 years of clinical and safety data, often in obese patients for bupropion (19). The most common adverse events with NB were nausea, constipation, and headache; these were generally mild or moderate and did not result in discontinuation in most participants. The seizure rate with NB in this trial (0.1%) is consistent with what has been previously described with bupropion (0.1% with doses up to 300 mg) (11). Consistent with the known pharmacology of bupropion (11), there was minimal change in blood pressure from baseline and a 1 bpm increase in pulse rate with NB. Importantly, the normal diurnal variation was maintained; nocturnal lowering in blood pressure is considered an important predictor of cardiovascular outcomes (20-21). Weight loss was correlated with reduced blood pressure in both groups, indicating that reductions can be expected for individuals experiencing meaningful weight loss with NB. NB was not associated with increased depression, depressed mood, or suicidality in this patient population (which excluded patients with current or recent major depressive disorder or suicidality).

Dysregulation of CNS reward pathways, in a manner similar to what is observed with addictive disorders, may contribute to the pathophysiology of obesity (22). Individually, bupropion and naltrexone are used to treat nicotine dependence (bupropion) and alcohol and opioid dependence (naltrexone). The effects of bupropion and naltrexone in treating addictive disorders may partly explain the increased ability of participants to control eating and avoid responding to food cravings in this study, as well as a previous study of NB32 (13). It is possible that these effects of naltrexone/bupropion on food cravings, which are a common barrier to adherence to a hypocaloric diet and may contribute to the failure of lifestyle modification in some individuals (23), facilitated adherence to the prescribed mild hypocaloric diet in this study.

Limitations of this study include a population composed mainly of middle-aged white females and a completion rate of 54% across all treatment groups. These are common in Phase 3 obesity trials (24-25). However, the high proportion of women in this trial represents the population most likely to receive weight loss pharmacotherapy (26). Although most metabolic parameters were normal at baseline and participants with diabetes were excluded, treatment effects favoring NB were observed in these parameters. Early study discontinuations may have influenced results in the mITT-LOCF population; however, sensitivity analyses were strongly supportive. Although the primary efficacy analyses were conducted at week 28, comparable findings at week 56 support the efficacy of NB32 over one year. Lastly, assessing the impact of NB on major cardiovascular events when the background rate is low, as evidenced by the single event in this trial, is challenging. A large, randomized, placebo-controlled trial evaluating major cardiovascular events with NB therapy is ongoing (Light Study, clinicaltrials.gov Identifier NCT01601704).

Overall, NB32 was generally well tolerated and produced clinically meaningful weight loss with corresponding improvements in some markers of cardiometabolic risk, weight-related quality of life, and control of eating, without evidence of negative effects on depression or suicidality. The addition of combination pharmacotherapies like NB to diet and lifestyle modification facilitate clinically meaningful weight loss, which may favorably impact obesity-associated comorbid conditions. This study suggests that NB32 has the potential to become a useful agent in the treatment of obesity and related health conditions.

### COR-II Study Group Members

Caroline Apovian, MD, Nutrition and Weight Management Center (Boston, Massachusetts); Louis Aronne, MD, Comprehensive Weight Control Program (New York, New York); Bruce Berwald, MD, Radiant Research, Inc. (Saint Louis, Missouri); Brian Bortnick, MD, Comprehensive NeuroScience, Inc. (Atlanta, Georgia); Susan Braun, MD, Lovelace Scientific Resources (Phoenix, Arizona); Robert Buynak, MD, Northwest Indiana Center for Clinical Research (Valparaiso, Indiana); Joseph Cleaver, MD, The Cooper Institute (Dallas, Texas); Martin Conway, MD, Lovelace Scientific Resources (Phoenix, Arizona); Milissa Cooper, DO, HOPE Research Institute, (Phoenix, Arizona); Neil Dubin, MD, Patient Priority (Cincinnati, Ohio); Steven Folkerth, MD, Clinical Research Center of Nevada (Las Vegas, Nevada); Martin Fritzhand, MD, Patient Priority (Cincinnati, Ohio); Forrest Hanke, MD, Trover Center for Clinical Studies (Madisonville, Kentucky); Jonathan G.A. Henry, MD, Summit Research Network (MI), Inc. (Okemos, Michigan); Lawrence Koehler, MD, Wells Institute for Health Awareness (Kettering, Ohio); Burton Lazar, MD, The Portland Clinic (Portland, Oregon); Michael T. Levy, MD, Behavioral Medical Research (Staten Island, New York); Norman Lunde, MD, Twin Cities Clinical Research (Brooklyn Center, Minnesota); Richard Mills, MD, Palmetto Medical Research (Mt. Pleasant, South Carolina); Nadar Oskooilar, MD, PhD, Pharmacology Research Institute (Newport Beach, California); Troy Oxner, DO, HealthStar Research (Hot Springs, Arkansas); Sanford Plevin, MD, Suncoast Clinical Research (Palm Harbor, Florida); Anthony Puopolo, MD, Milford Emergency Associates, Inc. (Milford, Massachusetts); George Raad, MD, Metrolina Medical Research (Charlotte, North Carolina); Domenica Rubino, MD, George Washington University (Washington DC); Nathan Segall, MD, Clinical Research Atlanta (Stockbridge, Georgia); Stephan C. Sharp, MD, Clinical Research Associates, Inc. (Nashville, Tennessee); Timothy Smith, MD, Mercy Health Research (Saint Louis, Missouri); Phillip Snell, MD, Mountain View Clinical Research (Greer, South Carolina); Joseph Soufer, MD, Chase Medical Research, LLC (Waterbury, Connecticut); Christopher Still, DO, Geisinger Medical Center (Danville, Pennsylvania); Paul Tung, MD, Endocrinology and Diabetes Consultants (Dover, New Hampshire); James Vogt, MD, HOPE Research Institute (Phoenix, Arizona); Claire Waltman, MD, Summit Research Network, Inc. (Seattle, Washington); Kevin Wingert, MD, Sierra Medical Research (Fresno, California); Holly Wyatt, MD, Center for Human Nutrition (Denver, Colorado); Douglas Young, MD, Northern California Research (Carmichael, California); Douglas Zmolek, MD, Central New York Clinical Research (Manlius, New York).

### Previous presentations

Portions of this study were presented at the XI International Conference on Obesity 2010 (Stockholm, Sweden), the American College of Cardiology Conference 60th Annual Scientific Session and i2 summit 2011 (New Orleans, Louisiana, USA), the Obesity Society Annual Meetings in 2009 (Washington DC) and 2010 (San Diego, California, USA), and the American Diabetes Association Annual Meeting 2011 (San Diego, California, USA).
