# Supplementary material for: A Randomized, Phase 3 Trial of Naltrexone SR/Bupropion SR on Weight and Obesity-related Risk Factors (COR-II)
Source: Obesity (Silver Spring). 2013 Feb 14;21(5):935–43. doi: 10.1002/oby.20309 (PMC3739931; doi:10.1002/oby.20309)
Supplement: Supplementary file 1 [file oby0021-0935-SD1.pdf]

Supplementary Appendix

This appendix has been provided by the authors to give readers additional information about their work.

Table of Contents

Section 1. Eligibility Criteria 2
Section 2. IWQOL-Lite Subscales 4
Section 3. Control of Eating Questionnaire 5
Section 4. Systolic / Diastolic Blood Pressure and Pulse Rate Outlier Analysis 8
Section 5. Relationship of Weight Loss with Vital Signs 9
Section 6. References 10

ScholarOne, 375 Greenbrier Drive, Charlottesville, VA, 22901

Section 1. Eligibility Criteria

Inclusion Criteria:

- Female and male subjects, 18 to 65 years of age
- Have body mass index (BMI) ≥30 and ≤45kg/m^2 for subjects with uncomplicated obesity, and BMI of ≥27 and ≤45kg/m^2 for subjects with obesity and controlled hypertension and/or dyslipidemia
    - Normotensive (systolic ≤140 mm Hg; diastolic ≤90 mm Hg). Anti-hypertensive medications are allowed with the exception of alpha-adrenergic blockers, and clonidine.
    - Medical regimen must be stable for at least 6 weeks prior to randomization
    - Medications for treatment of dyslipidemia are allowed as long as medical regimen has been stable for at least 6 weeks prior to randomization
- Free of any opioid medication for at least 7 days prior to randomization
- No clinically significant abnormality of serum albumin, blood urea nitrogen, creatinine, bilirubin, sodium, potassium, chloride, calcium or phosphorus
- ALT and AST within 2.5x upper limit of normal (ULN)
- No clinically significant abnormality of hematocrit, white blood cell count, white cell differential, or platelets
- Fasting glucose <126 mg/dL on no hypoglycemic agents, fasting triglycerides <400 mg/dL
- No clinically significant abnormality on urinalysis
- TSH within normal limits or normal T3, if TSH is below normal limits
- Negative serum pregnancy test in women of child bearing potential
- Negative urine drug screen
- IDS-SR scores <2 on items 5 (sadness), 6 (irritability), 7 (anxiety/tension) and 18 (suicidality), and IDS-SR total score is <30
- If woman of child bearing potential, must be non-lactating and agree to use effective contraception throughout the study period and 30 days after discontinuation of study drug
- Able to comply with all required study procedures and schedule
- Able to speak and read English
- Willing and able to give written informed consent

Exclusion Criteria:

- Obesity of known endocrine origin (e.g., untreated hypothyroidism, Cushing's syndrome)
- Serious medical condition (including but not limited to renal or hepatic insufficiency; Class III or IV congestive heart failure, history of angina pectoris, myocardial infarction, claudication, or acute limb ischemia within the previous 6 months; lifetime history of stroke)
- History of malignancy within the previous 5 years, with exception of non-melanoma skin cancer or surgically cured cervical cancer
- A lifetime history a serious psychiatric illness, including lifetime history of bipolar disorder, schizophrenia or other psychosis, bulimia, or anorexia nervosa
- Current serious psychiatric illness including severe personality disorder (e.g. borderline or antisocial), current severe major depressive disorder, recent (previous 6 months) suicide attempt, current active suicidal ideation or recent hospitalization due to psychiatric illness
- A response to Bipolar Disorder questions indicating the presence of Bipolar Disorder
- In need of medications for the treatment of a psychiatric disorder (with the exception of short-term insomnia) within the previous 6 months prior to randomization
- History of drug or alcohol abuse or dependence (with the exception of nicotine dependence) within 1 year prior to study participation.
- Type I or Type II diabetes mellitus
- Screening ECG with a QTc interval (Bazett's formula) >450 msec (men) and >470 msec (women) or the presence of any clinically significant cardiac abnormalities, including but not limited to patterns consistent with myocardial ischemia, electrolyte abnormalities, or atrial or ventricular dysrhythmia or significant conduction abnormalities

ScholarOne, 375 Greenbrier Drive, Charlottesville, VA, 22901

- Excluded concomitant medications: any psychotropic agents (including antipsychotic, antidepressant, anxiolytic, mood stabilizer, anticonvulsant or agents used for the treatment of Attention Deficit Disorder) with the exception of low dose benzodiazepine or hypnotic agents for the treatment of insomnia (up to 2 mg lorazepam/day or equivalent dose of benzodiazepine or hypnotic agent); any anorectic or weight loss agents; any over-the-counter dietary supplements with psychoactive, appetite or weight effects; alpha-adrenergic blockers; dopamine agonists; clonidine; coumadin; theophylline; cimetidine; oral corticosteroids; cholestyramine, cholestypol, Depo-Provera®; smoking cessation agents; regular use of opioid or opioid-like analgesics and anti-tussives.
- History of surgical or device (e.g. gastric banding) intervention for obesity
- History of seizures of any etiology, or of predisposition to seizures (e.g., history of cerebrovascular accident, head trauma with ≥5 minutes loss of consciousness, concussion symptoms lasting ≥15 minutes, brain surgery, skull fracture, subdural hematoma, or febrile seizures)
- History of treatment with bupropion, or naltrexone within the preceding 12 months
- History of hypersensitivity or intolerance to bupropion or naltrexone
- Initiation or discontinuation of tobacco products including inhaled tobacco (such as cigarettes, cigars, pipes, etc), chewing tobacco or snuff in the 3 months prior to randomization or planned during study participation. Use of nicotine replacement products (nicotine gum, patch etc) during study participation is not allowed.
- Use of drugs, herbs, or dietary supplements believed to significantly affect body weight or participation in a weight loss management program within one month prior to randomization
- Loss or gain of more than 4.0 kilograms within 3 months prior to randomization
- Pregnant or breast-feeding women or planning to become pregnant during the study period or within 30 days of discontinuing study drug
- Planned surgical procedure that can impact the conduct of the study
- Use of investigational drug, device or procedure within previous 30 days
- Participation in any previous clinical trial sponsored by Orexigen Therapeutics
- Any condition which in the opinion of the investigator makes the subject unsuitable for inclusion in this study
- Investigators, study personnel, sponsor representatives and their immediate families.

ScholarOne, 375 Greenbrier Drive, Charlottesville, VA, 22901

Section 2. IWQOL-Lite Subscales

Subscale [a, b]	Week 28			Week 56 [c]		
	Placebo	NB32	P-value	Placebo	NB32	P-value
**Physical function**						
Baseline	69.9 ± 19.9	69.9 ± 20.2		69.9 ± 20.1	69.8 ± 20.1	
Change	+8.0 ± 0.7	+13.1 ± 0.5	<0.001	+8.2 ± 0.8	+14.1 ± 0.6	<0.001
**Self-esteem**						
Baseline	56.8 ± 24.5	55.4 ± 25.7		57.1 ± 24.6	54.7 ± 25.1	
Change	+6.6 ± 1.0	+12.1 ± 0.7	<0.001	+7.3 ± 1.1	+13.8 ± 0.8	<0.001
**Sexual life**						
Baseline	76.5 ± 24.7	75.6 ± 26.7		76.4 ± 24.8	75.6 ± 27.0	
Change	+5.6 ± 0.9	+8.9 ± 0.7	0.003	+6.0 ± 1.0	+10.0 ± 0.7	<0.001
**Public distress**						
Baseline	87.5 ± 16.5	85.9 ± 18.4		87.6 ± 16.4	86.4 ± 18.2	
Change	+3.2 ± 0.6	+4.4 ± 0.4	0.087	+3.2 ± 0.6	+5.1 ± 0.4	0.005
**Work**						
Baseline	87.6 ± 16.1	86.2 ± 17.5		87.7 ± 16.1	86.4 ± 17.6	
Change	+4.4 ± 0.6	+5.4 ± 0.5	0.173	+3.8 ± 0.7	+5.4 ± 0.5	0.055

[a] Data are for the mITT-LOCF population, where the last observation on study drug was carried forward. Baseline values are mean ± SD; change values are LS mean ± SE.
[b] IWQOL-Lite scores are based on a scale from 0-100 where a score of 0-70 indicates severe impairment, 72-79 indicates moderate impairment, and 80-87 indicates mild impairment[1]
[c] Week 56 data are weighted as described in the statistical analyses section.

ScholarOne, 375 Greenbrier Drive, Charlottesville, VA, 22901

Section 3. Control of Eating Questionnaire

Item [Score: 0 to 100] [a, b]	Placebo	NB32 [c]	P-value
**1. How hungry have you felt? [Not at all hungry/Extremely hungry]**			
Baseline	59.0 ± 16.9	58.5 ± 18.7	
Week 8 change	-9.1 ± 1.0	-15.7 ± 0.7	<0.001
Week 28 change	-6.6 ± 1.0	-10.7 ± 0.7	<0.001
Week 56 change	-8.0 ± 1.0	-9.7 ± 0.8	0.148
**2. How full have you felt? [Not at all full/Extremely full]**			
Baseline	60.3 ± 19.2	60.6 ± 19.8	
Week 8 change	-0.9 ± 0.9	+1.7 ± 0.7	0.011
Week 28 change	-1.8 ± 0.8	+0.6 ± 0.6	0.022
Week 56 change	-2.0 ± 0.9	-0.9 ± 0.7	0.337
**3. How strong was your desire to eat sweet foods? [Not at all strong/Extremely strong]**			
Baseline	62.6 ± 27.3	59.9 ± 27.2	
Week 8 change	-11.9 ± 1.1	-18.9 ± 0.9	<0.001
Week 28 change	-10.6 ± 1.1	-13.3 ± 0.8	0.054
Week 56 change	-10.0 ± 1.2	-11.2 ± 0.9	0.409
**4. How strong was your desire for non-sweet tasty foods (French fries, potato chips, hamburgers, pizza)? [Not at all strong/Extremely strong]**			
Baseline	65.6 ± 22.1	64.3 ± 22.7	
Week 8 change	-12.8 ± 1.1	-15.4 ± 0.8	0.049
Week 28 change	-9.7 ± 1.1	-12.5 ± 0.8	0.031
Week 56 change	-10.0 ± 1.2	-12.6 ± 0.9	0.070
**5. How happy have you felt? [Not at all happy/Extremely happy]**			
Baseline	71.3 ± 15.9	70.7 ± 16.5	
Week 8 change	-2.1 ± 0.7	+0.3 ± 0.5	0.007
Week 28 change	-3.1 ± 0.7	-0.7 ± 0.5	0.006
Week 56 change	-2.6 ± 0.8	-0.2 ± 0.6	0.017
**6. How anxious have you felt? [Not at all anxious/Extremely anxious]**			
Baseline	28.9 ± 22.2	28.0 ± 21.7	
Week 8 change	+2.4 ± 1.0	+1.8 ± 0.8	0.662
Week 28 change	+5.2 ± 1.0	+2.9 ± 0.8	0.076
Week 56 change	+5.3 ± 1.1	+4.3 ± 0.8	0.456
**7. How alert have you felt? [Not at all alert/Extremely alert]**			
Baseline	72.6 ± 17.0	72.3 ± 17.6	
Week 8 change	-0.7 ± 0.8	+0.8 ± 0.6	0.125
Week 28 change	-2.1 ± 0.8	0.0 ± 0.6	0.029
Week 56 change	-1.7 ± 0.8	0.0 ± 0.6	0.090
**8. How contented have you felt? [Not at all contented/Extremely contented]**			
Baseline	71.6 ± 19.0	71.6 ± 19.4	
Week 8 change	-0.7 ± 0.8	+1.6 ± 0.6	0.019
Week 28 change	-2.4 ± 0.8	+0.2 ± 0.6	0.011
Week 56 change	-2.1 ± 0.9	-1.1 ± 0.7	0.380
**9. During the last 7 days, how often have you had food cravings? [Not at all/Very often]**			
Baseline	59.2 ± 24.2	58.7 ± 24.6	
Week 8 change	-10.1 ± 1.2	-17.3 ± 0.9	<0.001
Week 28 change	-7.6 ± 1.2	-12.3 ± 0.9	0.001
Week 56 change	-8.3 ± 1.3	-12.2 ± 0.9	0.012

ScholarOne, 375 Greenbrier Drive, Charlottesville, VA, 22901

Item [Score: 0 to 100]	Placebo	NB32	P-value
**10. How strong have any food cravings been? [Not at all strong/Extremely strong]**			
Baseline	58.5 ± 24.5	56.4 ± 25.6	
Week 8 change	-9.1 ± 1.2	-15.2 ± 0.9	<0.001
Week 28 change	-5.9 ± 1.2	-10.5 ± 0.9	0.002
Week 56 change	-6.8 ± 1.3	-9.3 ± 1.0	0.113
**11. How difficult has it been to resist any food cravings? [Not at all difficult/Extremely difficult]**			
Baseline	58.2 ± 26.7	58.6 ± 26.8	
Week 8 change	-11.7 ± 1.2	-19.8 ± 0.9	<0.001
Week 28 change	-9.2 ± 1.2	-14.4 ± 0.9	<0.001
Week 56 change	-9.9 ± 1.3	-13.8 ± 1.0	0.014
**12. How often have you eaten in response to food cravings? [Not at all/After every one]**			
Baseline	57.0 ± 24.4	57.0 ± 24.3	
Week 8 change	-12.5 ± 1.1	-18.8 ± 0.8	<0.001
Week 28 change	-10.7 ± 1.1	-14.7 ± 0.8	0.004
Week 56 change	-11.8 ± 1.2	-11.8 ± 0.9	0.992
**How often have you had cravings for the following:**			
**13. Chocolate or chocolate flavored foods? [Not at all/Extremely often]**			
Baseline	55.6 ± 30.0	52.1 ± 30.1	
Week 8 change	-9.8 ± 1.2	-16.6 ± 0.9	<0.001
Week 28 change	-9.7 ± 1.2	-11.6 ± 0.9	0.177
Week 56 change	-7.9 ± 1.3	-8.8 ± 1.0	0.558
**14. Other sweet foods (cakes, pastries, chocolate, etc)? [Not at all/Extremely often]**			
Baseline	51.8 ± 28.7	49.8 ± 28.5	
Week 8 change	-10.3 ± 1.1	-17.2 ± 0.8	<0.001
Week 28 change	-9.2 ± 1.1	-13.0 ± 0.9	0.006
Week 56 change	-9.5 ± 1.2	-11.3 ± 0.9	0.219
**15. Fruit or fruit juice? [Not at all/Extremely often]**			
Baseline	42.0 ± 26.1	40.4 ± 26.4	
Week 8 change	-1.3 ± 1.2	+0.1 ± 0.9	0.358
Week 28 change	-4.0 ± 1.2	+0.2 ± 0.9	0.004
Week 56 change	-4.0 ± 1.3	+1.6 ± 0.9	<0.001
**16. Dairy foods (cheese, yogurts, milk, etc)? [Not at all/Extremely often]**			
Baseline	49.2 ± 26.7	47.2 ± 26.7	
Week 8 change	-5.3 ± 1.1	-5.0 ± 0.8	0.855
Week 28 change	-5.3 ± 1.1	-5.0 ± 0.8	0.849
Week 56 change	-5.1 ± 1.2	-4.4 ± 0.9	0.644
**17. Starchy foods (bread, rice, pasta, etc.)? [Not at all/Extremely often]**			
Baseline	55.4 ± 27.3	53.2 ± 26.2	
Week 8 change	-10.8 ± 1.1	-12.9 ± 0.8	0.121
Week 28 change	-8.5 ± 1.1	-12.7 ± 0.8	0.002
Week 56 change	-9.6 ± 1.2	-12.4 ± 0.9	0.051
**18. Tasty foods that are not sweet (French fries, potato chips, burgers, pizza, etc.)? [Not at all/Extremely often]**			
Baseline	60.3 ± 24.5	60.7 ± 25.1	
Week 8 change	-13.9 ± 1.1	-15.6 ± 0.9	0.225
Week 28 change	-10.1 ± 1.1	-12.8 ± 0.9	0.057
Week 56 change	-10.5 ± 1.3	-13.0 ± 1.0	0.110

ScholarOne, 375 Greenbrier Drive, Charlottesville, VA, 22901

Item [Score: 0 to 100]	Placebo	NB32	P-value
**19. Generally, how difficult has it been to control your eating? [Not at all difficult/Extremely difficult]**			
Baseline	62.0 ± 23.5	61.9 ± 24.1	
Week 8 change	-13.7 ± 1.1	-24.7 ± 0.8	<0.001
Week 28 change	-11.1 ± 1.1	-18.3 ± 0.9	<0.001
Week 56 change	-11.3 ± 1.2	-15.9 ± 0.9	0.002
**20. Which one food makes it most difficult for you to control eating?** [d]			
**21. How difficult has it been to resist eating this food during the last 7 days? [Not at all difficult/Extremely difficult]**			
Baseline	66.2 ± 24.3	66.7 ± 24.3	
Week 8 change	-12.6 ± 1.3	-16.9 ± 1.0	0.005
Week 28 change	-10.6 ± 1.2	-13.9 ± 0.9	0.029
Week 56 change	-13.0 ± 1.3	-13.2 ± 1.0	0.873

[a] Data are for the mITT-LOCF population, where the last observation on study drug was carried forward. Baseline values are mean ± SD; change values are LS mean ± SE.
[b] Participants were instructed to base their responses on their experience within the previous 7 days. A food craving is defined as a strong urge to eat a particular food or drink.
[c] Week 56 data are weighted as described in the statistical analyses section.
[d] Answers to this question varied by participant and are not reported here.

ScholarOne, 375 Greenbrier Drive, Charlottesville, VA, 22901

Section 4. Systolic / Diastolic Blood Pressure and Pulse Rate Outlier Analysis (Safety Analysis Set)

	Placebo (N=492) n (%)	NB32 (N=992) n (%)
Subjects with ≥1 postbaseline measurement reported	464 (94.3%)	874 (88.1%)
**Blood Pressure**		
Subjects with ≥2 consecutive values above baseline[1]		
Systolic ≥10 mm Hg	83 (17.9%)	203 (23.2%)
Diastolic ≥5 mm Hg	148 (31.9%)	331 (37.9%)
Subjects with ≥2 consecutive values above threshold[1,2]		
Systolic ≥160 mm Hg	0 (0.0%)	2 (0.2%)
Diastolic ≥100 mm Hg	1 (0.2%)	2 (0.2%)
**Pulse Rate**		
Subjects with ≥2 consecutive values above baseline[1]		
≥5 bpm	203 (43.8%)	451 (51.6%)
≥10 bpm	89 (19.2%)	219 (25.1%)
Subjects with ≥2 consecutive values above threshold[1,2]		
≥100 bpm	2 (0.4%)	10 (1.1%)

[1]Or single measurement above threshold if final blood pressure or pulse rate measurement.

[2]Subjects with blood pressure or pulse rate value above stated threshold at baseline were excluded from analysis.

ScholarOne, 375 Greenbrier Drive, Charlottesville, VA, 22901

Section 5. Relationship of Weight Loss with Vital Signs

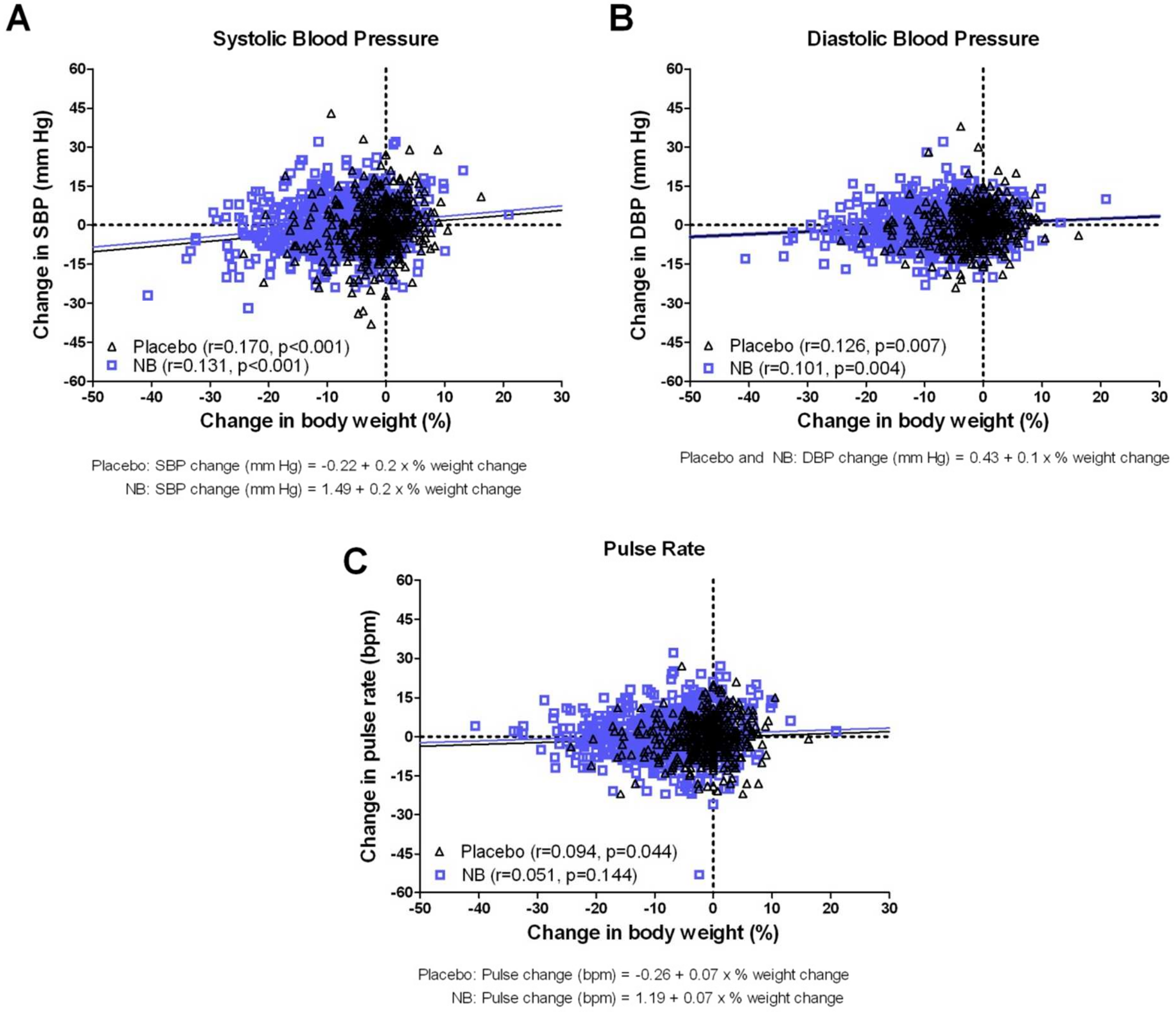

Percent change in body weight from baseline to Week 56 endpoint and change from baseline to Week 56 endpoint in (A) systolic blood pressure, (B) diastolic blood pressure, and (C) pulse rate were plotted for each individual participant. Data are for the mITT-LOCF unweighted population, where all NB participants were pooled together regardless of re-randomization status (Placebo N=456, NB N=825). Linear regression models were used to fit equations for each treatment group. Statistical testing was conducted on model parameters to determine the most parsimonious model fit. A common slope model for the two treatment groups was used for both systolic blood pressure and pulse rate versus percent change in body weight, while a common slope and intercept model for the two treatment groups was used for diastolic blood pressure versus percent change in body weight.

ScholarOne, 375 Greenbrier Drive, Charlottesville, VA, 22901

Section 6. References

Crosby RD, Kolotkin RL, Williams GR. An integrated method to determine meaningful changes in health-related quality of life. *J Clin Epidemiol.* Nov 2004;57(11):1153-1160.

ScholarOne, 375 Greenbrier Drive, Charlottesville, VA, 22901
